# Supplementary material for: Regional sequencing collaboration reveals persistence of the T12 Vibrio cholerae O1 lineage in West Africa
Source: eLife. 2021 Jun 18;10:e65159. doi: 10.7554/eLife.65159 (PMC8213408; doi:10.7554/eLife.65159)
Supplement: Supplementary file 1. [file elife-65159-supp1.docx]

## **Supplementary File 1**

**Supplementary File 1A**. Quinolone susceptibility and mutations in the quinolone-resistance determining regions of DNA gyrase and topoisomerase IV in *Vibrio cholerae* O1.

| **Isolate name** | **Disc Diffusion** | | **Mutations** | | | | | | | |
| --- | --- | --- | --- | --- | --- | --- | --- | --- | --- | --- |
|  | *Nalidixic acid* | *Ciprofloxacin* | *GyrA* | | | | *ParC* | *GyrB* | | |
|  |  |  | *S83* | *S171* | *S202* | *S412* | *S85* | *T142* | *D159* | *V665* |
| CMR_VC_06 | Resistant | Susceptible | I | A | A | R | L | S | E | I |
| CMR_VC_07 | Resistant | Susceptible | I | A | A | R | L | S | E | I |
| CMR_VC_14 | Resistant | Susceptible | I | A | A | R | L | S | E | I |
| CMR_VC_16 | Resistant | Susceptible | I | A | A | R | L | S | E | I |
| CMR_VC_22 | Resistant | Susceptible | I | A | A | R | L | S | E | I |
| CMR_VC_28 | Resistant | Susceptible | I | A | A | R | L | S | E | I |
| CMR_VC_29 | Resistant | Susceptible | I | A | A | R | L | S | E | I |
| CMR_VC_30 | Resistant | Susceptible | I | A | A | R | L | S | E | I |
| CMR_VC_31 | Resistant | Susceptible | I | A | A | R | L | S | E | I |
| CMR_VC_33 | Resistant | Susceptible | I | A | A | R | L | S | E | I |
| CMR_VC_34 | Resistant | Susceptible | I | A | A | R | L | S | E | I |
| CMR_VC_44 | Resistant | Susceptible | I | A | A | R | L | S | E | I |
| CMR_VC_48 | Resistant | Susceptible | I | A | A | R | L | S | E | I |
| CMR_VC_50 | Resistant | Susceptible | I | A | A | R | L | S | E | I |
| CMR_VC_56 | Resistant | Susceptible | I | A | A | R | L | S | E | I |
| CMR_VC_57 | Resistant | Susceptible | I | A | A | R | L | S | E | I |
| NER_003_2018 | Resistant | Susceptible | I | A | A | R | L | S | E | I |
| NER_004_2016 | Resistant | Susceptible | I | A | A | R | L | S | E | I |
| NER_004_2018 | Resistant | Susceptible | I | A | A | R | L | S | E | I |
| NER_005_2018 | Resistant | Susceptible | I | A | A | R | L | S | E | I |
| NER_023_2018 | Resistant | Susceptible | I | A | A | R | L | S | E | I |
| NER_024_2018 | Resistant | Susceptible | I | A | A | R | L | S | E | I |
| NER_028_2011 | Resistant | Susceptible | I | A | A | R | L | S | E | I |
| NER_055_2011 | Resistant | Susceptible | I | A | A | R | L | S | E | I |
| NER_071_2011 | Resistant | Susceptible | I | A | A | R | L | S | E | I |
| NER_088_2011 | Resistant | Susceptible | I | A | A | R | L | S | E | I |
| NER_109_2011 | Resistant | Susceptible | I | A | A | R | L | S | E | I |
| NER_116_2011 | Resistant | Susceptible | I | A | A | R | L | S | E | I |
| NER_132_2012 | Resistant | Susceptible | I | A | A | R | L | S | E | I |
| NER_205_2011 | -- | -- | I | A | A | R | L | S | E | I |
| NER_211_2018 | Resistant | Susceptible | I | A | A | R | L | S | E | I |
| NGA_001_2019 | -- | Susceptible | I | A | A | R | L | S | E | I |
| NGA_002_2019 | -- | -- | I | A | A | R | ND | S | E | I |
| NGA_016_2019 | -- | Susceptible | I | A | A | R | L | S | E | I |
| NGA_201_2018 | -- | Susceptible | I | A | A | R | L | S | E | I |
| NGA_205_2018 | -- | -- | I | A | A | R | ND | S | E | I |
| NGA_206_2018 | -- | -- | I | A | A | R | ND | S | E | I |
| NGA_220_2018 | -- | Susceptible | I | A | A | R | L | S | E | I |
| NGA_235_2018 | -- | Susceptible | I | A | A | R | L | S | E | I |
| NGA_236_2019 | -- | -- | I | A | A | R | ND | S | E | I |
| NGA_247_2018 | -- | Susceptible | I | A | A | R | L | S | E | I |
| NGA_248_2018 | -- | Susceptible | I | A | A | R | L | S | E | I |
| NGA_255_2018 | -- | Susceptible | I | A | A | R | L | S | E | I |
| NGA_263_2018 | -- | -- | I | A | A | R | ND | S | E | I |

S: Serine; I: Isoleucine; L: Leucine; A: Alanine; E: Glutamate; --: Not tested; ND: Not Determined.

No mutations in *parE* genes were observed compared to the reference peptide sequence (susceptible). The two non-O1 genomes (NGA_148_2019 and NGA_252_2019) were not included in this analysis.

**Supplementary File 1B.** Phenotypic antibiotic resistance profile versus genotypic profile.

| **Isolate** | **Beta-lactam** | | | **Quinolone** | | | **Phenicol** | | **Polymyxin** | | |
| --- | --- | --- | --- | --- | --- | --- | --- | --- | --- | --- | --- |
|  | *varG* | *AMC* | *AMP* | *CRP* | *NAL* | *CIP* | *catB9* | *CHL* | *almG* | *PMB* | *CST* |
| CMR_VC_06 | Pr | R | -- | Pr | R | S | Pr | S | Pr | R | R |
| CMR_VC_07 | Pr | R | -- | Pr | R | S | Pr | S | Pr | R | R |
| CMR_VC_14 | Pr | R | -- | Pr | R | S | Pr | S | Pr | R | R |
| CMR_VC_16 | Pr | R | -- | Pr | R | S | Pr | S | Pr | R | R |
| CMR_VC_22 | Pr | R | -- | Pr | R | S | Pr | S | Pr | R | R |
| CMR_VC_28 | Pr | R | -- | Pr | R | S | Pr | S | Pr | R | R |
| CMR_VC_29 | Pr | R | -- | Pr | R | S | Pr | S | Pr | R | R |
| CMR_VC_30 | Pr | R | -- | Pr | R | S | Pr | S | Pr | R | R |
| CMR_VC_31 | Pr | R | -- | Pr | R | S | Pr | S | Pr | R | R |
| CMR_VC_33 | Pr | R | -- | Pr | R | S | Pr | S | Pr | R | R |
| CMR_VC_34 | Pr | R | -- | Pr | R | S | Pr | S | Pr | R | R |
| CMR_VC_44 | Pr | R | -- | Pr | R | S | Pr | S | Pr | R | R |
| CMR_VC_48 | Pr | R | -- | Pr | R | S | Pr | S | Pr | R | R |
| CMR_VC_50 | Pr | R | -- | Pr | R | S | Pr | S | Pr | R | R |
| CMR_VC_56 | Pr | R | -- | Pr | R | S | Pr | S | Pr | R | R |
| CMR_VC_57 | Pr | R | -- | Pr | R | S | Pr | S | Pr | R | R |
| NER_003_2018 | Pr | R | -- | Pr | R | S | Pr | -- | Pr | -- | -- |
| NER_004_2016 | Pr | R | -- | Pr | R | S | Pr | -- | Pr | -- | -- |
| NER_004_2018 | Pr | R | -- | Pr | R | -- | Pr | -- | Pr | -- | -- |
| NER_005_2018 | Pr | R | -- | Pr | R | S | Pr | -- | Pr | -- | -- |
| NER_023_2018 | Pr | R | -- | Pr | R | S | Pr | -- | Pr | -- | -- |
| NER_024_2018 | Pr | R | -- | Pr | R | -- | Pr | -- | Pr | -- | -- |
| NER_028_2011 | Pr | R | -- | Pr | R | S | Pr | -- | Pr | -- | -- |
| NER_055_2011 | Pr | R | -- | Pr | R | S | Pr | -- | Pr | -- | -- |
| NER_071_2011 | Pr | R | -- | Pr | R | S | Pr | -- | Pr | -- | -- |
| NER_088_2011 | Pr | R | -- | Pr | R | S | Pr | -- | Pr | -- | -- |
| NER_109_2011 | Pr | R | -- | Pr | R | S | Pr | -- | Pr | -- | -- |
| NER_116_2011 | Pr | R | -- | Pr | R | S | Pr | -- | Pr | -- | -- |
| NER_132_2012 | Pr | R | -- | Pr | R | S | Pr | -- | Pr | -- | -- |
| NER_205_2011 | Pr | R | -- | Pr | R | S | Pr | -- | Pr | -- | -- |
| NER_211_2018 | Pr | R | -- | Pr | R | S | Pr | -- | Pr | -- | -- |
| NGA_001_2019 | Pr | -- | R | Pr | R | S | Pr | S | Pr | -- | -- |
| NGA_002_2019 | Pr | -- | I | Pr | R | S | Pr | S | Pr | -- | -- |
| NGA_016_2019 | Pr | -- | I | Pr | R | S | Pr | S | Pr | -- | -- |
| NGA_201_2018 | Pr | -- | I | Pr | R | S | Pr | S | Pr | -- | -- |
| NGA_205_2018 | Pr | -- | I | Pr | R | S | Pr | S | Pr | -- | -- |
| NGA_206_2018 | Pr | -- | R | Pr | R | S | Pr | S | Pr | -- | -- |
| NGA_220_2018 | Pr | -- | I | Pr | R | S | Pr | S | Pr | -- | -- |
| NGA_235_2018 | Pr | -- | I | Pr | R | S | Pr | S | Pr | -- | -- |
| NGA_247_2018 | Pr | -- | I | Pr | R | S | Pr | S | Pr | -- | -- |
| NGA_248_2018 | **Abs** | -- | I | Pr | R | S | Pr | S | **Abs** | -- | -- |
| NGA_255_2018 | Pr | -- | R | Pr | R | S | Pr | S | Pr | -- | -- |

### Pr: Gene present; Abs: Gene absent; R: Resistant isolate; S: Susceptible isolate; I: Intermediate; --: Not tested; AMC: amoxicillin-clavulanic acid; AMP: ampicillin; NAL: nalidixic acid; CHL: chloramphenicol; PMB: polymyxin B; CST: colistin. All samples had *tet(34)* present and were susceptible to tetracycline; all samples had *farA* present and were not tested for antibacterial free fatty acids. The following samples were not tested and thus not included in this analysis: NGA_236_2019, NGA_263_2018, NGA_148_2019 and NGA_252_2019.
