# Supplementary material for: Regional sequencing collaboration reveals persistence of the T12 Vibrio cholerae O1 lineage in West Africa
Source: eLife. 2021 Jun 18;10:e65159. doi: 10.7554/eLife.65159 (PMC8213408; doi:10.7554/eLife.65159)
Supplement: Transparent reporting form [file elife-65159-transrepform.docx]

***eLife’s* transparent reporting form**

We encourage authors to provide detailed information *within their submission* to facilitate the interpretation and replication of experiments. Authors can upload supporting documentation to indicate the use of appropriate reporting guidelines for health-related research (see [EQUATOR Network](http://www.equator-network.org/%20)), life science research (see the [BioSharing Information Resource](https://biosharing.org/)), or the [ARRIVE guidelines](http://www.plosbiology.org/article/info:doi/10.1371/journal.pbio.1000412) for reporting work involving animal research. Where applicable, authors should refer to any relevant reporting standards documents in this form.

If you have any questions, please consult our Journal Policies and/or contact us: [editorial@elifesciences.org](mailto:editorial@elifesciences.org).

**Sample-size estimation**

- You should state whether an appropriate sample size was computed when the study was being designed
- You should state the statistical method of sample size computation and any required assumptions
- If no explicit power analysis was used, you should describe how you decided what sample (replicate) size (number) to use

The second paragraph of the results section details how samples were selected for sequencing: we selected samples from the three countries represented, prioritizing good spread over time and space, as well as selecting samples near the country borders to capture possible cross-border transmissions. We did not perform an explicit power calculation, but our primary finding (that the T12 lineage was circulating in all three countries in 2018-2019) would not change with the addition of further samples and data.

Please outline where this information can be found within the submission (e.g., sections or figure legends), or explain why this information doesn’t apply to your submission:

**Replicates**

- You should report how often each experiment was performed
- You should include a definition of biological versus technical replication
- The data obtained should be provided and sufficient information should be provided to indicate the number of independent biological and/or technical replicates
- If you encountered any outliers, you should describe how these were handled
- Criteria for exclusion/inclusion of data should be clearly stated
- High-throughput sequence data should be uploaded before submission, with a private link for reviewers provided (these are available from both GEO and ArrayExpress)

Please outline where this information can be found within the submission (e.g., sections or figure legends), or explain why this information doesn’t apply to your submission:

We sequenced each sample once, using sufficient depth to ensure >100X coverage across >95% of the Vibrio cholerae genome. This data is available in Figure 2 – Source Data 1. Outliers include two samples that belong to a different serogroup (non-O1) and did not align to the O1 reference – these results were removed from the O1-specific phylogenetic analysis and are concordant with laboratory testing for Vibrio cholerae serogroup. Sequencing data was uploaded to SRA. Accession numbers are available in the “Data Availability” section in Methods.

**Statistical reporting**

- Statistical analysis methods should be described and justified
- Raw data should be presented in figures whenever informative to do so (typically when N per group is less than 10)
- For each experiment, you should identify the statistical tests used, exact values of N, definitions of center, methods of multiple test correction, and dispersion and precision measures (e.g., mean, median, SD, SEM, confidence intervals; and, for the major substantive results, a measure of effect size (e.g., Pearson's r, Cohen's d)
- Report exact p-values wherever possible alongside the summary statistics and 95% confidence intervals. These should be reported for all key questions and not only when the p-value is less than 0.05.

No applicable: Statistical tests were not performed in this study, as they were not required to comment on the Vibrio cholerae lineages circulating in West Africa in 2018-2019.

Please outline where this information can be found within the submission (e.g., sections or figure legends), or explain why this information doesn’t apply to your submission:

(For large datasets, or papers with a very large number of statistical tests, you may upload a single table file with tests, Ns, etc., with reference to sections in the manuscript.)

**Group allocation**

- Indicate how samples were allocated into experimental groups (in the case of clinical studies, please specify allocation to treatment method); if randomization was used, please also state if restricted randomization was applied
- Indicate if masking was used during group allocation, data collection and/or data analysis

Not applicable: experimental groups were not needed for the experimental outputs (high-throughput sequencing and AMR detection assays).

Please outline where this information can be found within the submission (e.g., sections or figure legends), or explain why this information doesn’t apply to your submission:

**Additional data files (“source data”)**

- We encourage you to upload relevant additional data files, such as numerical data that are represented as a graph in a figure, or as a summary table
- Where provided, these should be in the most useful format, and they can be uploaded as “Source data” files linked to a main figure or table
- Include model definition files including the full list of parameters used
- Include code used for data analysis (e.g., R, MatLab)
- Avoid stating that data files are “available upon request”

Please indicate the figures or tables for which source data files have been provided:

Case counts by epidemiological week for all three countries have been collated from publicly available data and are available as Figure 1 – Source Data 1. Locations and dates for the samples sequenced in this study are available as Figure 1 – Source Data 2.

Genome accessions used to create the tree in Figure 2 are provided in Figure 2 – Source Data 3, and annotations needed for this figure are available in Figure 1 – Source Data 2.

Code used to generate figures is provided as Source Code File 1. The full assembly pipeline used to generate sequence data from raw MinION data is available at: <https://github.com/HopkinsIDD/minion-vc>, as indicated in the manuscript text.
